# Fabrication, *in vitro* and *ex vivo* evaluation of proliposomes and liposomal derived gel for enhanced solubility and permeability of diacerein

**DOI:** 10.1371/journal.pone.0258141

**Published:** 2021-10-19

**Authors:** Hassan Shah, Asadullah Madni, Muhammad Abdur Rahim, Nasrullah Jan, Arshad Khan, Safiullah Khan, Abdul Jabar, Ahsan Ali

**Affiliations:** 1 Department of Pharmaceutics, Faculty of Pharmacy, The Islamia University of Bahawalpur, Bahawalpur, Punjab, Pakistan; 2 College of Pharmacy, University of Sargodha, Sargodha, Punjab, Pakistan; Bahauddin Zakariya University, PAKISTAN

## Abstract

The present study is associated with the development of proliposomes and liposomal derived gel for enhanced solubility and permeability of diacerein. Proliposomes were developed by thin film hydration method and converted into the liposomal derived gel using carbopol-934 as a gelling agent. Formulations with varied lecithin to cholesterol ratios were investigated to obtain the optimal size, entrapment efficiency, and enhanced *in vitro* dissolution. Dynamic light scattering analysis revealed the particle size and zeta potential in the range of 385.1±2.45–762.8±2.05 nm and -22.4±0.55–31.2±0.96mV respectively. Fourier transform infrared (FTIR) spectroscopic analysis depicted the physicochemical compatibility, powdered x-ray diffraction (PXRD) analysis predicted the crystalline nature of pure drug and its transition into amorphous form within formulation. The differential scanning calorimetry (DSC) demonstrated the thermal stability of the formulation. The *in vitro* drug release study using dialysis membrane displayed the enhanced dissolution of diacerein due to the presence of hydrophilic carrier (Maltodextrin) followed by sustained drug release due to the presence of lipid mixture (lecithin and cholesterol). *Ex vivo* permeation studies depicted 3.50±0.27 and 3.21±0.22 folds enhanced flux of liposomal gels as compared to control. The acute oral toxicity study showed safety and biocompatibility of the system as no histopathological changes in vital organs were observed. These results suggests that proliposomes and liposomal derived gel are promising candidates for the solubility and permeability enhancement of diacerein in the management of osteoarthritis.

## 1. Introduction

Over the few decades, considerable attention has been given to the development of novel drug delivery systems. The novel drug delivery systems aim to deliver the drug at the target site to meet the requirements of the body [1]. Novel drug delivery carriers used for targeted delivery of drugs include particulate systems, polymeric micelles, nano and microparticulate systems. Particulate carrier systems include micro and nanoparticles, lipid based systems and vesicular systems. Vesicular drug delivery systems (VDDS) are highly ordered assemblies consisting of one or more concentric bilayers formed due to the self-assembling of amphiphilic part by hydration. VDDS have attracted keen interest for targeted delivery of drugs because of their ability of local action at the site or organ and thereby lowering the drug concentration at the other sites in the body [2].

Among the various types of the vesicular drug delivery approaches, liposomes were discovered by A. D. Bangham in 1965 [3]. Liposomes provide a versatile platform for the delivery of hydrophilic and hydrophobic drugs due to their concentric nature. These are lipid bilayer spherical structured vesicles that deliver the drug by active or passive targeting that led the foundation for the targeted delivery to the specific area of the body [4]. Liposomes are the ideal carrier systems having similarities to that of the biological cell membrane for the delivery of therapeutic agents. As liposomes, mainly composed of phospholipids having amphiphilic nature with a hydrophilic head and hydrophobic tail. Upon their dispersion, the head portion interact with aqueous phase whereas the entanglement of hydrophobic tails takes place with one another. Therefore, for the last five decades, liposomes have been widely investigated and attracted keen interest for the biological and technological advantages as the optimal delivery system for biologically active substances, both *in vitro* and *in vivo* [5]. However, liposomes are the relatively unstable system that is manifested by physical and chemical instability [6].

To overcome these stability problems, the proliposomes (PLs) have been presented and reported initially in 1986, as dry, free flowing granular powders that form liposomes when dispersed in water. In PLs, the active drug and lipid mixture (phospholipid and cholesterol) were coated by a carrier that provided the free flowing characteristics, higher stability and solubility to the resultant vesicles compared to the conventional liposomes [7].

Phospholipids have been utilized to increase skin permeation and used in the dispersed form as penetration enhancers in the vesicular systems. The liposomal gel fabricated from phospholipids is recognized to have skin permeation enhancing ability as phospholipids integrate with the lipid covering of stratum corneum acting as penetration enhancers of drugs [8]. Moreover, it has been reported that drug loaded liposomal gel has better permeation than control gel [9].

Diacerein is a prodrug and is classified as a semi-synthetic derivative of anthraquinone, converted into Rhein (active metabolite) [10]. Diacerein is classified as British Classification System (BCS) class II with low solubility and high permeability [11]. Diacerein has limited aqueous solubility which results in low oral bioavailability (35–56%). Diacerein is a chondroprotective agent and is indicated for the management of osteoarthritis [12].

The PLs have been used for the solubility and permeability enhancement of certain drugs [13–15] and the present study is based on the solubility and permeability enhancement of diacerein through PLs and liposomal derived gel. Herein, the diacerein loaded proliposomes have been developed for oral delivery and liposomal gel for topical application for the management of osteoarthritis.

## 2. Material and methods

### 2.1. Materials

Diacerein was received as a research material donation from the Global Pharmaceuticals (Pvt) Ltd., Islamabad, Pakistan. Egg lecithin was purchased from Rongsheng Bio-Tech Co., Ltd., Shanghai, China. Soy lecithin was received as a gift from Lipoid, Switzerland, and cholesterol was purchased from AppliChem, Darmstadt Germany. Carbopol-934, maltodextrin (DE 16.5–19.5), triethanolamine, methanol and chloroform of HPLC grade were purchased from Sigma Aldrich, Chemie GmbH, Germany.

### 2.2. Preparation of proliposomes (PLs)

PLs containing diacerein were formulated by varied ratios of lipid (egg lecithin and soy lecithin) to cholesterol and fixed amounts of maltodextrin (Table 1). The thin layer hydration method reported by Nekkanti *et al*. was employed for the preparation of PLs with slight modifications [16]. Briefly, varied ratios of lipid and cholesterol and a fixed amount of diacerein (10mg) were dissolved in a 12 mL mixture of methanol and chloroform in the ratio of 1:2. The solution was poured into the round bottom flask containing maltodextrin. The drying of dispersion was done by rotary evaporator (Heidolph, Germany) at 600 mmHg and temperature at 65±2°C for 5 hours. The dried PL powder obtained was further dried in an oven (Memmert GmbH) at 45°C for 24 hours to achieve dried free flowing powder.

**Table 1:** Composition of diacerein loaded proliposomes of egg lecithin and soy lecithin.

Formulation code	Lipid: Cholesterol (75μmol)	Lipid (mg)	Cholesterol (mg)	Diacerein (mg)	Maltodextrin (mg)	Chloroform (mL)	Methanol (mL)
Egg lecithin based Proliposomes							
D1PL	7.5:2.5	42.63	7.23	10	225	8	4
D2PL	6.5:3.5	36.95	10.13	10	225	8	4
D3PL	5.5:4.5	31.26	13.02	10	225	8	4
D4PL	4.5:5.5	25.58	15.92	10	225	8	4
D5PL	3.5:6.5	19.89	18.81	10	225	8	4
D6PL	2.5:7.5	14.21	21.71	10	225	8	4
Soy lecithin based Proliposomes							
D7PL	7.5:2.5	36.22	7.23	10	225	8	4
D8PL	6.5:3.5	31.39	10.13	10	225	8	4
D9PL	5.5:4.5	26.56	13.02	10	225	8	4
D10PL	4.5:5.5	21.73	15.92	10	225	8	4
D11PL	3.5:6.5	16.90	18.81	10	225	8	4
D12PL	2.5:7.5	12.07	21.71	10	225	8	4

### 2.3. Evaluation of PL powder

#### 2.3.1. Determination of yield (%)

The PL powders were collected and weighed accurately to determine the amount of weight loss during handling and formulation. The percentage yield was determined by the following formula [17];

Yield(%)=Practicalyield/Theoraticalyield×100 (1)

#### 2.3.2. Flow properties of PL powder

The flow properties of the PL powder were measured through an angle of repose. The fix funnel method was employed for the determination of the angle of repose. The PL powder was allowed to fall on paper through a funnel at 10 cm above the surface of the paper. The formula for angle of repose is given as [18, 19]:

Tanθ=2H/D (2)

“θ” denotes the angle of repose, “H” is the height of cone formed and “D” is the diameter of the cone

#### 2.3.3. Scanning Electron Microscopic (SEM) analysis

The morphology and surface characteristics of diacerein loaded PLs were observed under Field Emission Scanning Electron Microscope (FESEM, JSM-5910, JEOL, Japan). The PL powder was placed on a piece of the electro-conductive chip of silicon over the top of the aluminium stubs. The samples were coated on the stubs and examined at different magnifications [20].

#### 2.3.4. Fourier Transform Infrared Spectroscopic (FTIR) analysis

Fourier transform infrared spectroscopic analysis has been performed to evaluate the drug-excipient interaction. Diacerein, cholesterol, egg lecithin, soy lecithin and maltodextrin were analyzed by FTIR (Bruker, Tensor 27 series, Germany). The samples were analyzed in the range of 4000 to 500 cm^−1^. The samples were analyzed by zinc selenium attenuated total reflectance (ATR) mode set at 16 scans per sample [21].

#### 2.3.5. Powdered x-ray diffraction (PXRD) analysis

PXRD analysis of diacerein, cholesterol, egg lecithin, soy lecithin, physical mixture and PLs powder formulations (D5PL, D11PL) were performed to analyze the effect of encapsulation on the crystalline properties. The x-ray diffractograms were obtained by using PXRD diffractometer (Bruker Axs, D8 Advance, Germany) with CuKα radiation by applying the monochromator voltage of 45 kV and current 40 mA. The range for 2θ diffraction angle was from 05–70 per minute [19].

#### 2.3.6. Thermal stability by Differential Scanning Calorimetric (DSC) analysis

The thermal stability of diacerein, physical mixtures and optimized formulations was estimated through DSC analysis. The sample was accurately weighed in aluminium pans and covered with aluminium lid. The thermograms were recorded at a scan rate of 10°C min^-1^ by heating samples from 25°C-600°C [22].

### 2.4. Preparation of liposomes

The liposomes were prepared by hydration of PL powder formulations. Briefly, PL powder equivalent to 3 mg of drug was dispersed in quantity sufficient to 5 mL of phosphate buffer of pH 6.8 to obtain liposomal suspension in eppendorf tubes and vortexed for 1–2 minutes in the vortex mixer (My Lab, SeoLin Bioscience, Seoul, Korea). The formed liposomes were stored in an air-tight container for further evaluation [13].

### 2.5. Evaluation of liposomes

#### 2.5.1. Analysis of particle size, polydispersity index and zeta potential

The analysis of particle size, polydispersity index and zeta potential are useful tools for the determination of size, size distribution and colloidal stability. These parameters were determined by the dynamic light scattering technique using zeta sizer and zeta potential analyzer (ZS-90, Malvern Instruments, Malvern, Worcestershire, UK) [16].

#### 2.5.2. Transmission electron microscopy

The morphology of liposomes after hydration of PLs was carried out by transmission electron microscopy. The samples of liposomes were applied on the grid and the suitable images were taken at different magnifications [20].

#### 2.5.3. Entrapment efficiency (%EE) and drug loading capacity (%LC)

The percent amount of drug entrapped by the liposomal formulation was determined by an indirect method. The liposomal dispersion was centrifuged at 12,000 rpm (Ultracentrifuge machine, Sigma, Germany) for 45 minutes. The supernatant layer was taken, further, centrifugation was performed and resuspended in PBS. The procedure was repeated thrice (*n* = 3) and then the samples were observed through a UV/Visible spectrophotometer (IRMECO, U2020, Germany) at 258.7 nm. The percent entrapment efficiency and drug loading capacity of liposomes and proliposomes were calculated by the following formula [23]:

$$Encapsulation\;efficiency\;(\%)=\frac{\begin{array}{c} Total\;amount\;ofdrug-Unentrapped\\ drug \end{array}}{Total\;amount\;of\;drug}\times 100$$ (3)

$$Drug\;loading\;capacity\;(\%)=\frac{\begin{array}{c} Total\;amount\;of\;drug-Unentrapped\\ drug \end{array}}{Total\;mass\;of\;proliposomes/Liposomes}\times 100$$ (4)

#### 2.5.4. *In vitro* release studies

The *in vitro* drug release studies of all the PL derived liposomal formulations were carried out by the USP type II dissolution apparatus (paddle) (Pharma test W00 4895, Hainburg, Germany). A weighted amount of liposomal formulation equivalent to 2 mg of diacerein was taken in dialysis membrane (12–14 KDa) (Medicell Membranes Ltd, UK) and immersed in 200 mL of phosphate buffer (pH 1.2 and 6.8). The dissolution studies were carried out at pre-set conditions of 37±2°C temperature and 70 rpm stirring speed. The samples were taken at the intervals of 0.25, 0.5, 0.75, 1, 1.5, 2, 3, 4, 5, 6, 9, 12 and 16 hrs. In each sample, 3 mL of dissolution medium was taken and substituted with the 3 mL fresh dissolution medium to maintain the desired sink conditions. The samples obtained were filtered and analyzed at λ_max_ of 258.7 nm. The drug concentrations were determined and plotted versus time for the percent release rate by using DDsolver.xla (Add-In of MS-Excel). The regression equation was useful for the determination of unknown concentrations of diacerein in the liposomal dispersion [24].

#### 2.5.5. Kinetics of drug release

The release kinetics indicated the mechanism and order of the drug release from the PLs formulations. The dissolution data were applied to the zero order, first order, Higuchi and Korsmeyer-Peppas kinetic models. The correlation coefficient (R^2^) for the various kinetic models and the release exponent (n) values for the Korsmeyer-Peppas models were determined by the DDsolver.xla.

### 2.6. Preparation of liposomal gel

The PL derived liposomal gels were prepared by taking an amount equivalent to 2 mg drug. 1% w/v Carbopol-934 solution was prepared and stirred by an overhead stirrer [25] (Euro Star Digital Ika Werke^®^, Staufen, Germany) at 1000 rpm for 2 hours. The weighed amount of liposomal suspension was added dropwise to the carbopol-934 solution with continuous stirring. The final volume was made up to 100 mL with the distilled water.

Whereas, the 1% w/v Carbopol-934 based drug loaded gel was developed and was considered as control drug gel. In brief, 1% w/v Carbopol-934 aqueous dispersion was prepared and after soaking, the dispersion was stirred by an overhead stirrer at 1000 rpm for 2 hours. 2 mg diacerein was incorporated in the carbopol-934 dispersion with continuous stirring and in the final step the pH of the gels was adjusted with 0.9% w/v triethanolamine aqueous solution. The prepared liposomal gels (D5PL-G, D11PL-G and control gel) were kept in an air-tight container for further use.

### 2.7. Evaluation of Liposomal gel

#### 2.7.1. Permeation studies by using excised Wistar rat skin

The permeability of diacerein loaded gel (control gel) and liposomal gels (D5PL-G and D11PL-G) were evaluated by Franz diffusion cell (Perme Gear, Inc. No: 4G-01-00-15-12) with a volume capacity of 12 mL and surface area of the opening of 1.76 cm^2^ using hairless, excised rat skin. The skin of the Wistar rat obtained was cleaned with isopropyl alcohol for the removal of residual fat and impurities. Phosphate buffer of pH 7.4 was used as a medium in the receptor compartment. The temperature at 35±0.5°C and stirring speed of 300 rpm for 24 hours were selected as experimental conditions. The samples were taken at predefined time intervals and an equal volume of the phosphate buffer was added to the Franz diffusion cell to maintain the sink conditions.

The absorbance of the samples after regular intervals of time was analyzed by a UV-Visible spectrophotometer (IRMECO, U2020, Germany). The graph was plotted between the amount of diacerein permeated across the Wistar rat skin through liposomal gel and time. The various parameters of the drug permeability such as flux and enhancement ratio were calculated [24, 26].

The apparent permeability was calculated by the following formula;

J=Kp×Ci (5)

Where “*Kp*” is the coefficient of permeability, “*J*” is flux or apparent permeability and “*Ci*” is Drug concentration (initial) in the compartment.


Kp=Va×Da/AT×Dd (6)

“*Va*” volume of receptor compartment, “*A*” area of Franz cell opening, “*T*” is time, “*Da*” is the concentration of drug in receptor compartment and “*Dd*” is the concentration in the donor compartment. Enhancement ratio can be given as:

E.R=J(formulation)/J(control) (7)

### 2.8. Acute oral toxicity study

The acute toxicity study were performed to evaluate the safety and biocompatibility of the excipients used for the fabrication of proliposomes. Blank proliposomes were administered orally to the test animals and the study was performed according to Organization for Economic Cooperation and Development (OECD) guidelines. The study protocols have been reviewed and approved by Pharmacy Research and Ethics Committee (Ref No: 57-2019/PREC [27–29].

#### 2.8.1. Selection of animal species

For the acute oral toxicity study, Wistar rats weighing 180–220 gm were selected.

#### 2.8.2. Housing and feeding conditions

The temperature in the experimental animal room was kept at 22°C (±3°C). The relative humidity was at least 30% and preferably did not exceed 70%. Artificial lighting was maintained with the sequence being 12 hours light, 12 hours dark. For feeding, conventional laboratory diets were used within an unlimited supply of drinking water.

#### 2.8.3. Preparation of animals

The Wistar rats were marked to permit the individual identification, and kept in their separate cages for 5 days before dosing to allow for acclimatization to the laboratory conditions.

#### 2.8.4. Number of animals and dose administered

The 15 Wistar rats were selected and divided into three groups. Distilled water was administered to group-I (control group), whereas, egg lecithin based liposomes and soy lecithin based liposomes were orally administered to group-II and group-III in the dose of 2000mg/kg body weight accordingly. Animals were fasted over-night before dosing, food but not withheld water. Following the period of fasting, the Wistar rats were weighed and the test substance was administered.

#### 2.8.5. Observation of animals

Animals were observed individually after dosing during the first 30 minutes, periodically during the first 24 hours, with special attention given during first 4 hours, and daily thereafter, for a total 14 days. The observation of test animals (Wistar rats) was carefully monitored for signs of illness, any visible skin toxicity/irritation, and mortality.

#### 2.8.6. Euthanization of animals

At 15^th^ day, the animals were anesthetized with (xylazine and ketamine) in order to relieve any distress/discomfort, blood samples were collected from Wistar rats for blood biochemistry and then euthanized for histopathological examination of vital organs.

### 2.9. Statistical analysis

The one-tailed *t*-test statistical analysis was applied on *in vitro* release and permeation studies and the *p*-value was calculated to evaluate the effect of lipid concentration and lipid used of different sources (egg and soy lecithin).

## 3. Results and discussion

The present study was designed to prepare the PLs to enhance the solubility, improve the stability and accomplish the sustained release of diacerein. The PLs were successfully prepared by thin film hydration method.

### 3.1. Evaluation of proliposomal powder

#### 3.1.1. Determination of yield (%)

The yield (%) of the PL formulations (D1PL-D12PL) is an important parameter that represents the recovery of the formulation components after the final formulation was prepared (Table 2). The percentage yield of egg lecithin based PL formulations (D1PL-D6PL) was in the range of 77.82±1.53% to 87.46±1.78% and percentage yield of soy lecithin based formulations (D7PL-D12PL) was in the range of 59.15± 2.56% to 87.50± 1.50% represented the optimal recovery of formulation components.

**Table 2:** Evaluation of different parameters of proliposomal formulations.

Formulation code	Particle Size (nm)	PDI	Zeta Potential (mV)	Yield (%)	Entrapment Efficiency (%)	Loading Capacity (Liposomes)(%)	Loading Capacity (Proliposomes) (%)	Angle of Repose (θ)
D1PL	385.1±2.45	0.330±0.05	-24.3±0.55	83.80±1.41	57.92±1.02	8.29±1.03	1.79±0.04	25.51±1.65
D2PL	415.0±3.19	0.302±0.04	-27.7±0.60	78.65±2.81	59.84±1.99	8.27±1.01	1.73±0.06	29.38±1.40
D3PL	443.3±4.10	0.278±0.02	-29.8±0.91	87.46±1.78	65.33±0.94	7.95±0.57	2.13±0.04	26.53±1.21
D4PL	626.5±3.15	0.272±0.06	-22.6±0.61	77.82±1.53	76.26±1.45	8.79±0.74	2.50±0.05	25.40±1.04
D5PL	679.2±2.75	0.377±0.04	-31.2±0.96	84.19±1.69	86.13±1.19	6.43±0.85	3.12±0.07	25.31±1.27
D6PL	762.8±2.05	0.382±0.03	-28.1±0.53	79.26±2.35	72.43±0.72	7.87±0.71	2.42±0.04	25.39±1.49
D7PL	412.9±3.11	0.242±0.16	-22.4±0.55	59.15±2.56	62.47±1.64	7.99±0.85	1.28±0.03	24.90±1.80
D8PL	473.4±2.79	0.358±0.03	-29.2±0.55	76.16±1.51	65.49±0.81	6.87±0.84	1.95±0.04	25.42±1.99
D9PL	506.3±2.40	0.332±0.02	-25.6±0.86	83.15±1.59	67.29±1.30	7.45±1.07	2.16±0.03	29.24±1.27
D10PL	524.6±2.96	0.259±0.01	-29.4±0.83	81.09±2.18	71.48±1.31	8.24±0.71	2.37±0.04	27.77±1.27
D11PL	591.3±2.47	0.407±0.01	-30.9±0.78	87.50±1.50	78.66±0.89	7.19±1.03	2.73±0.05	26.01±1.38
D12PL	634.7±2.00	0.310±0.01	-24.8±0.64	86.67±2.79	74.33±0.84	7.65±0.74	2.60±0.04	24.85±1.56

All values are expressed as mean ± *SD* (*n* = 3).

#### 3.1.2. Flow properties of PL powder

The flow properties of PLs powder were assessed from the angle of repose and were in the range of 24.85±1.56^ο^-29.38±1.40^ο^ (Table 2). The smaller angles of repose values indicated non-cohesiveness among the particles of formulation, whereas, the gradual increase in the values might indicate the increase in cohesiveness among the particles due to more internal friction. The angle of repose of all proliposomal powder was less than 30 indicating good flow properties [19].

#### 3.1.3. Scanning Electron Microscopic (SEM) analysis

**Fig 1:**
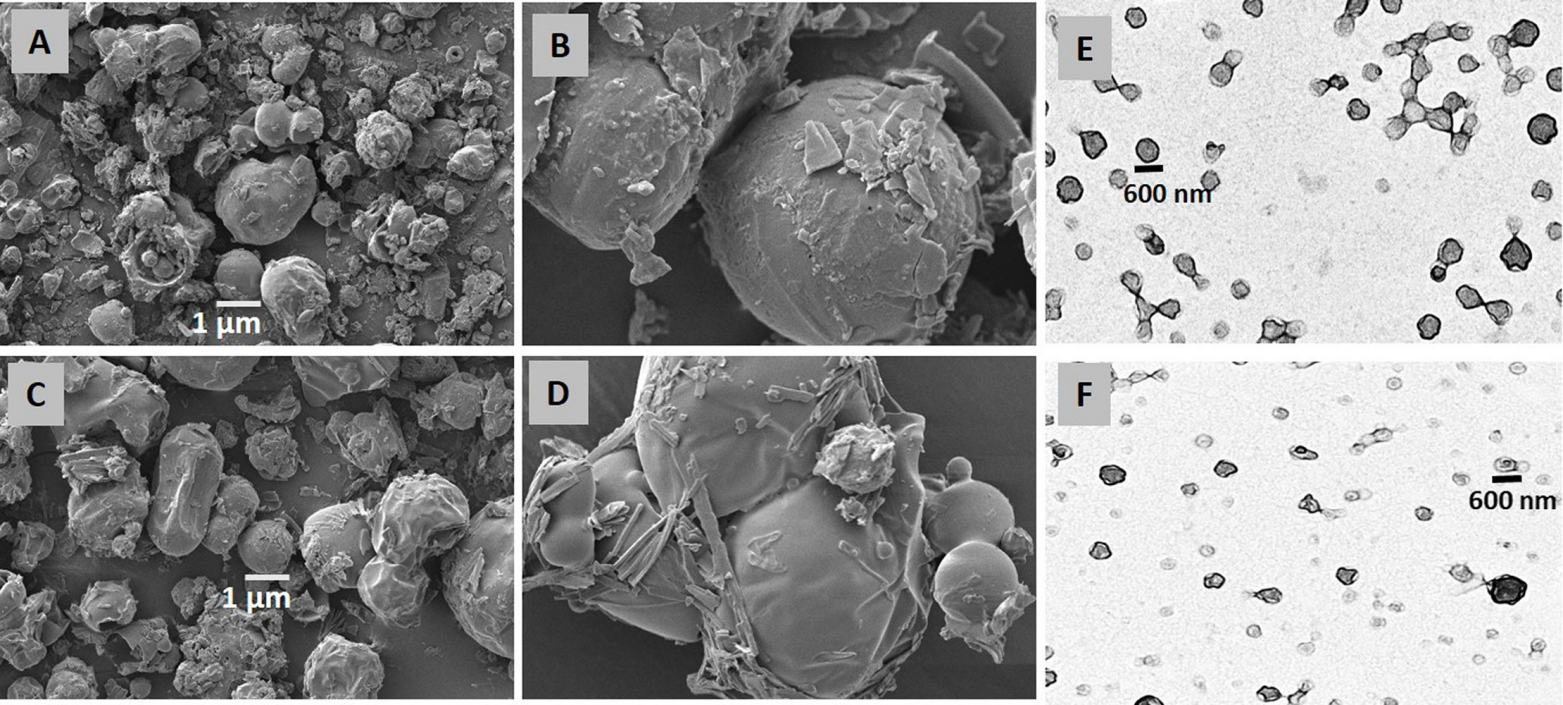
Scanning electron microscopic images of D5PL (A, B) and D11PL (C, D) and transmission electron microscopic images of D5PL (E) and D11PL (F).

Scanning electron microscopic analysis was used to determine the surface morphology of the developed PLs. The SEM analysis illustrated the spherical shape of PLs with the amorphous surface as shown in Fig 1. Moreover, SEM images revealed the porous nature with a size range of 1 μm. The maltodextrin possesses porous nature with a high surface area which enables it to be used as an efficient carrier for the lipid coating. Similar effects of maltodextrin as a carrier were also reported by Gurrapu, A., et al., 2012 [30].

#### 3.1.4. Fourier Transform Infrared Spectroscopic (FTIR) analysis

**Fig 2:**
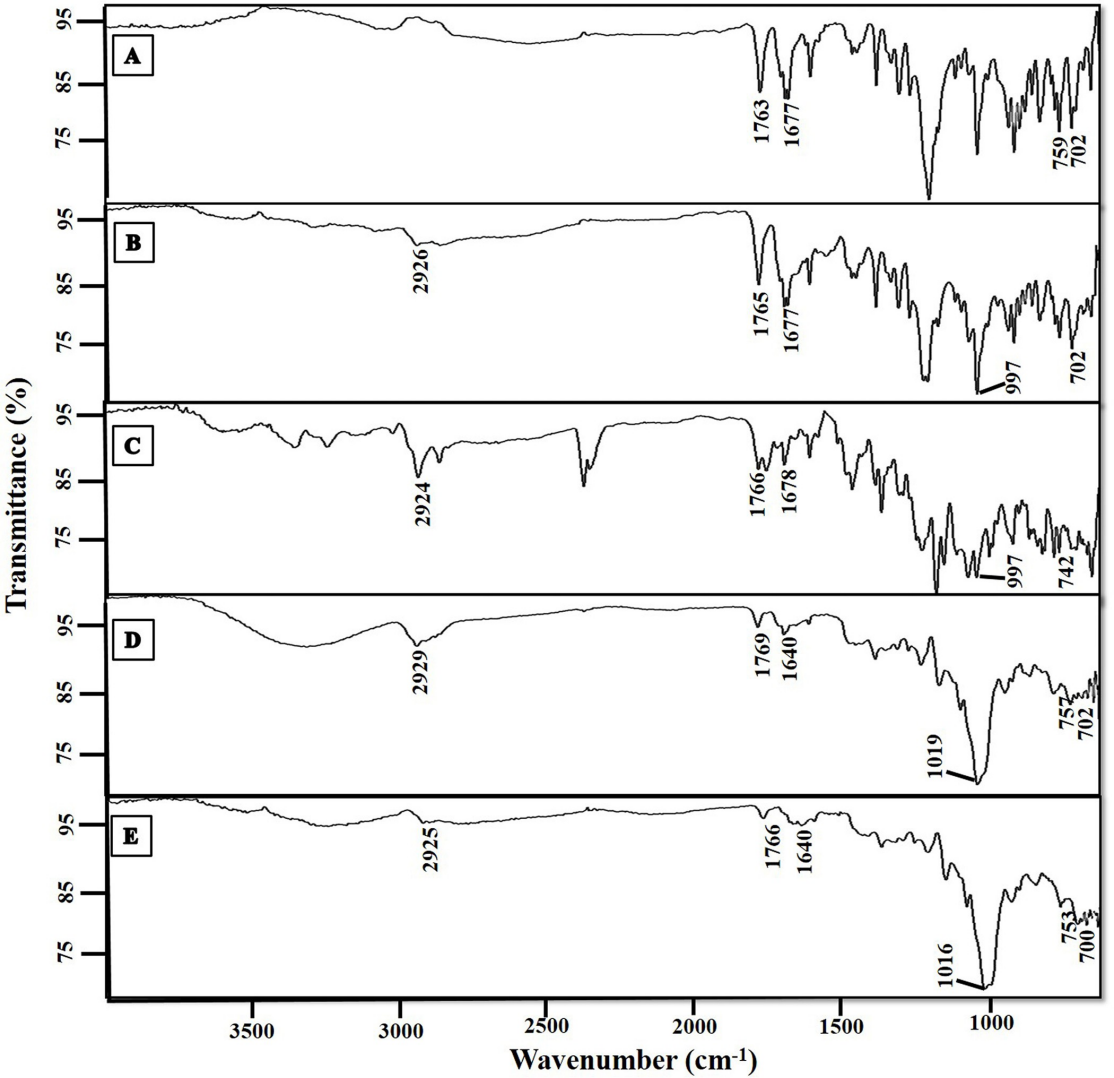
FTIR analysis of diacerein (A), egg lecithin based physical mixture (B), soy lecithin based physical mixture (C), D5PL (D) and D11PL (E).

The FTIR spectra of diacerein showed characteristic stretching peaks at 1763 cm^-1^ (ester group), at 1677 cm^-1^ (keto group), at 759 cm^-1^ (m-substituted benzene ring) and at 702 cm^-1^ (benzene ring) respectively (shown in Fig 2) [31, 32].

The FTIR spectra of cholesterol, maltodextrin, egg lecithin and soy lecithin are also attached (S1 Fig in S1 Data). The egg lecithin based physical mixture showed peaks at 2926 cm^-1^ (2921 cm^-1^ of lipid), 1765 cm^-1^ (1763 cm^-1^ of diacerein), 1677 cm^-1^ (1677 cm^-1^ of diacerein), at 997 cm^-1^ (997 cm^-1^ of maltodextrin) and at 702 cm^-1^ (702 cm^-1^ of diacerein). Likewise, the soy lecithin based physical mixture showed peaks at 2924 cm^-1^ (2921cm^-1^ of lipid), 1766 cm^-1^ (1763 cm^-1^ of diacerein), at 1678 cm^-1^ (1677 cm^-1^ of diacerein), at 997 cm^-1^ (997 cm^-1^ of maltodextrin) and at 742 cm^-1^ (753 cm^-1^ of diacerein).

The egg lecithin based formulation (D5PL) showed peaks at 2929 cm^-1^ (2921 cm^-1^ of lecithin), 1769 cm^-1^ (1763 cm^-1^ of diacerein), at 1019 cm^-1^ (997 cm^-1^ of maltodextrin as peak shifted to slightly high intensity) at 757 cm^-1^ (759 cm^-1^ of diacerein) and at 702 cm^-1^ (702 cm^-1^ of diacerein) respectively. Likewise, the soy lecithin based formulation (D11PL) showed peaks at 2925 cm^-1^ (2921 cm^-1^ of lecithin), 1766 cm^-1^ (1763 cm^-1^ of diacerein), at 1016 cm^-1^ (997 cm^-1^ of maltodextrin as peak shifted to slightly high intensity) 753 cm^-1^ (759 cm^-1^ of diacerein), and at 700 cm^-1^ (702 cm^-1^ of diacerein) respectively.

The characteristics peaks of diacerein in PL formulations suggested the efficient entrapment of diacerein within the system and indicated the physicochemical stability of the excipients used for the PL formulations.

#### 3.1.5. Powdered x-ray diffraction (PXRD) analysis

**Fig 3:**
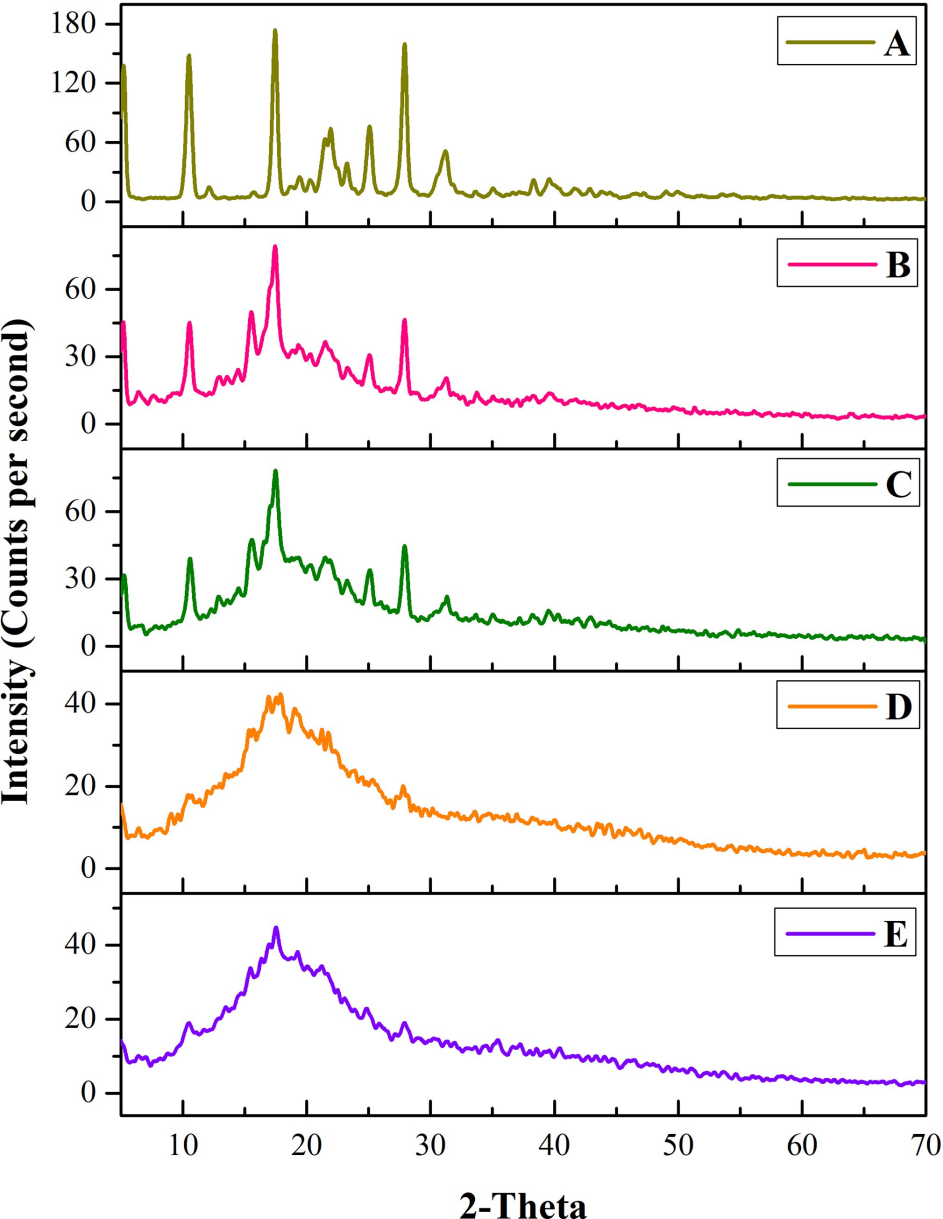
Powder x-ray diffractogram of diacerein (A), egg lecithin based physical mixture (B), soy lecithin based physical mixture (C), D5PL (D) and D11PL (E).

The powdered x-ray diffractogram of diacerein represented peaks at 10.54°, 17.42°, and 27.92° which depicts their crystalline nature (shown in Fig 3) [33]. The sharp characteristics peaks of diacerein were also prominent in the egg lecithin based physical mixture at 10.58°, and 17.42° and soy lecithin based physical mixture at 10.50°, and 17.50°. Whereas, the crystalline peaks were absent in PL formulations (D5PL and D11PL), which predicts the conversion of the drug from crystalline to amorphous form. The diacerein peaks were not observed in the formulation (D5PL and D11PL), clearly indicating the modification in physical state from crystalline to amorphous state.

#### 3.1.6. Thermal stability by Differential Scanning Calorimetric (DSC) analysis

**Fig 4:**
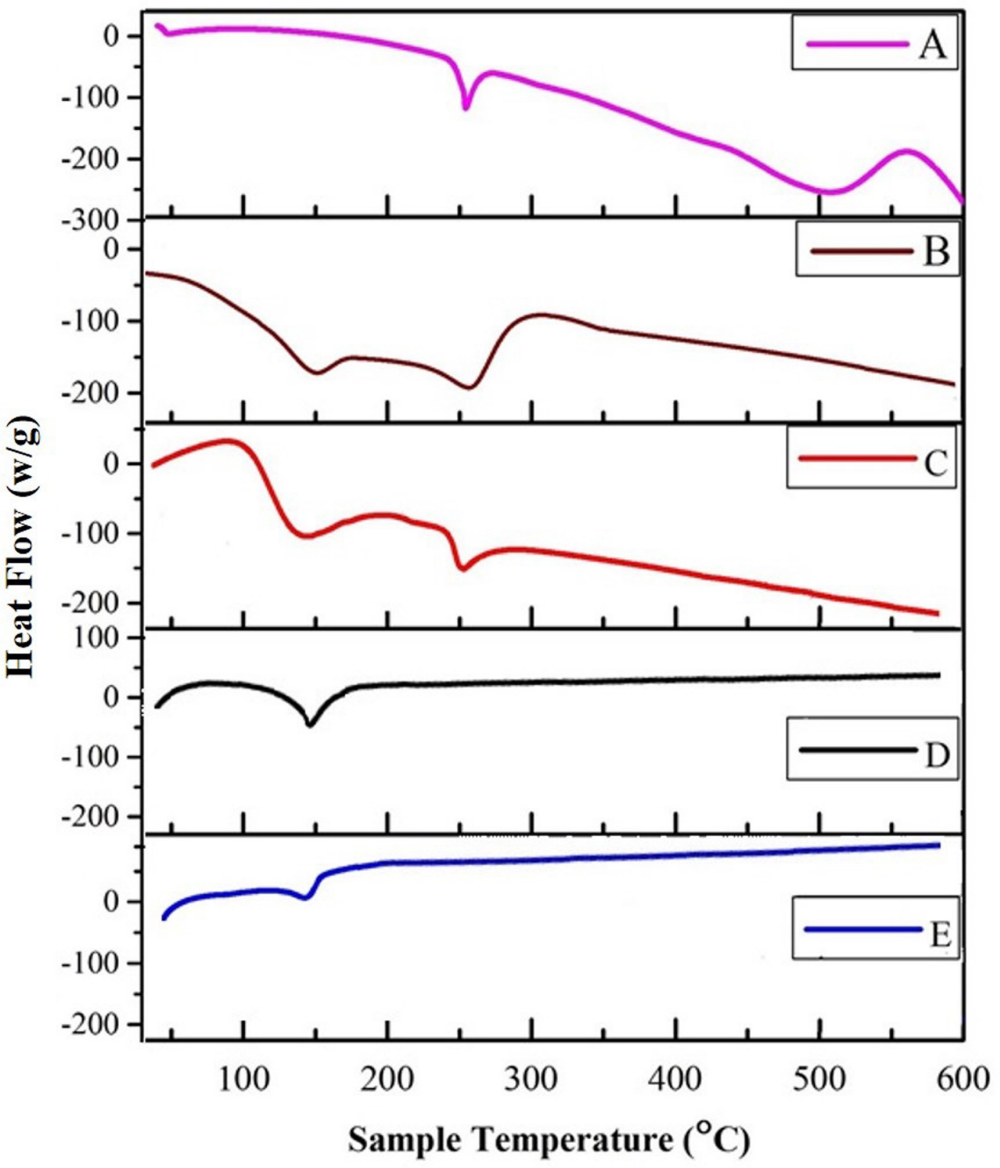
DSC of diacerein (A), egg lecithin based physical mixture (B), soy lecithin based physical mixture (C), D5PL (D) and D11PL (E).

The thermal analysis and physical state of diacerein in proliposomal formulations were ascertained from the DSC thermograms. The DSC thermograms of diacerein, their physical mixtures and formulations (D5PL and D11PL) are shown in Fig 4. Whereas, the thermograms of cholesterol, maltodextrin, egg and soy lecithin are also mentioned (S2 Fig in S1 Data). The thermograms of diacerein (Fig 4A) showed a sharp endothermic peak at 250°C correspondings to the melting point of diacerein. The egg and soy lecithin based physical mixtures also showed a peak of cholesterol and diacerein. Moreover, in the formulations (Fig 4D and 4E), the absence of characteristics peak over the range of melting point of diacerein is an indication of the successful entrapment of drug within the vesicles and transformation of the crystalline form of the drug into an amorphous form.

### 3.2. Evaluation of liposomes

#### 3.2.1. Analysis of particle size, polydispersity index and zeta potential

The particle size of egg lecithin (D1PL-D6PL) and soy lecithin (D7PL-D12PL) based liposomal formulations were in the range of 385.1±2.45 nm-762.8±2.05 nm and 412.9±3.11nm-634.7±2.00 nm respectively (Table 2). The particle size was significantly influenced by the cholesterol concentration. As the cholesterol concentration was increased, the particle size was also increased, due to an increase in the thickness of the vesicle bilayer. This finding was also in good agreement with previous studies conducted by Velpula, A., et al., 2012 [34].

The polydispersity index (PDI) values of all liposomal formulations were less than 0.45, which indicates the mono-dispersity of PL derived liposomes. The zeta potential values of egg lecithin (-22.6 ± 0.61 mV to -31.2 ± 0.96 mV) and soy lecithin (-22.4 ± 0.55 mV to -30.9 ± 0.78 mV) based formulations (Table 2) indicates the good stability of the liposomal system. The results of zeta potential are also supported by the study reported by Bobbala, S.K.R. and P.R. Veerareddy, 2012 [19]. The particle size of egg lecithin based liposomes showed higher particle size as compared to soy lecithin based liposomes. The lipid:cholesterol ratios showed a direct and significant effect on the particle size analysis (*p*<0.05).

#### 3.2.2. Transmission electron microscopy

The transmission electron microscopic (TEM) images of the liposomes after hydration are shown in Fig 1E and 1F. The TEM images revealed the 600 nm average size of the liposomes after hydration. The TEM images showed the spherical morphology of the liposomes which is in accordance with the size of the liposomes achieved by DLS technique.

#### 3.2.3. Entrapment efficiency and loading capacity

The EE of egg lecithin based liposomal formulations was in the range of 57.92±1.02% to 86.13±1.19% (Table 2). Likewise, the EE of soy lecithin based liposomal formulations (D7PL–D12PL) ranged from 62.47±1.64% to 78.66±0.89%.

Cholesterol is one of the important components used in the fabrication of proliposomes and liposomes and has a direct effect on the EE. Upon increasing the cholesterol contents the EE increases, as it provides vesicular stability to the liposomes. The aforementioned behavior is associated with a certain limit of the cholesterol concentration (D1PL-D5PL) and (D7PL-D11PL). But, when cholesterol content is above this limit, the entrapment efficiency decreases as in the case of D6PL and D12PL. The reason may be due to competition between the diacerein and cholesterol within the vesicle and the system will retain the increased cholesterol concentration due to its stable nature. A similar effect is also reported by Ning, M.-Y., et al., 2005 [35]. The lipid:cholesterol ratios showed a direct and significant effect on the entrapment efficiency (*p*<0.05).

Whereas, the LC is the percentage of drug successfully loaded in the liposomes. The LC of the formulations was in the range of 6.43±0.85–8.79±0.74 as shown in Table 2. The minimum loading capacity was observed for D5PL (6.43±0.85) and the maximum for D4PL (8.79±0.74). The LC of egg lecithin based liposomes and proliposomes showed higher LC as compared to soy lecithin based liposomes and proliposomes. The LC of proliposomes was in the range of 1.28±0.03–3.12±0.07. The minimum loading capacity were observed for D7PL (1.28±0.03), and the maximum was observed for D5PL (3.12±0.07). The lipid:cholesterol ratios showed a significant effect on the LC of both liposomes and proliposomes (*p*<0.05).

#### 3.2.4. *In vitro* release study

**Fig 5:**
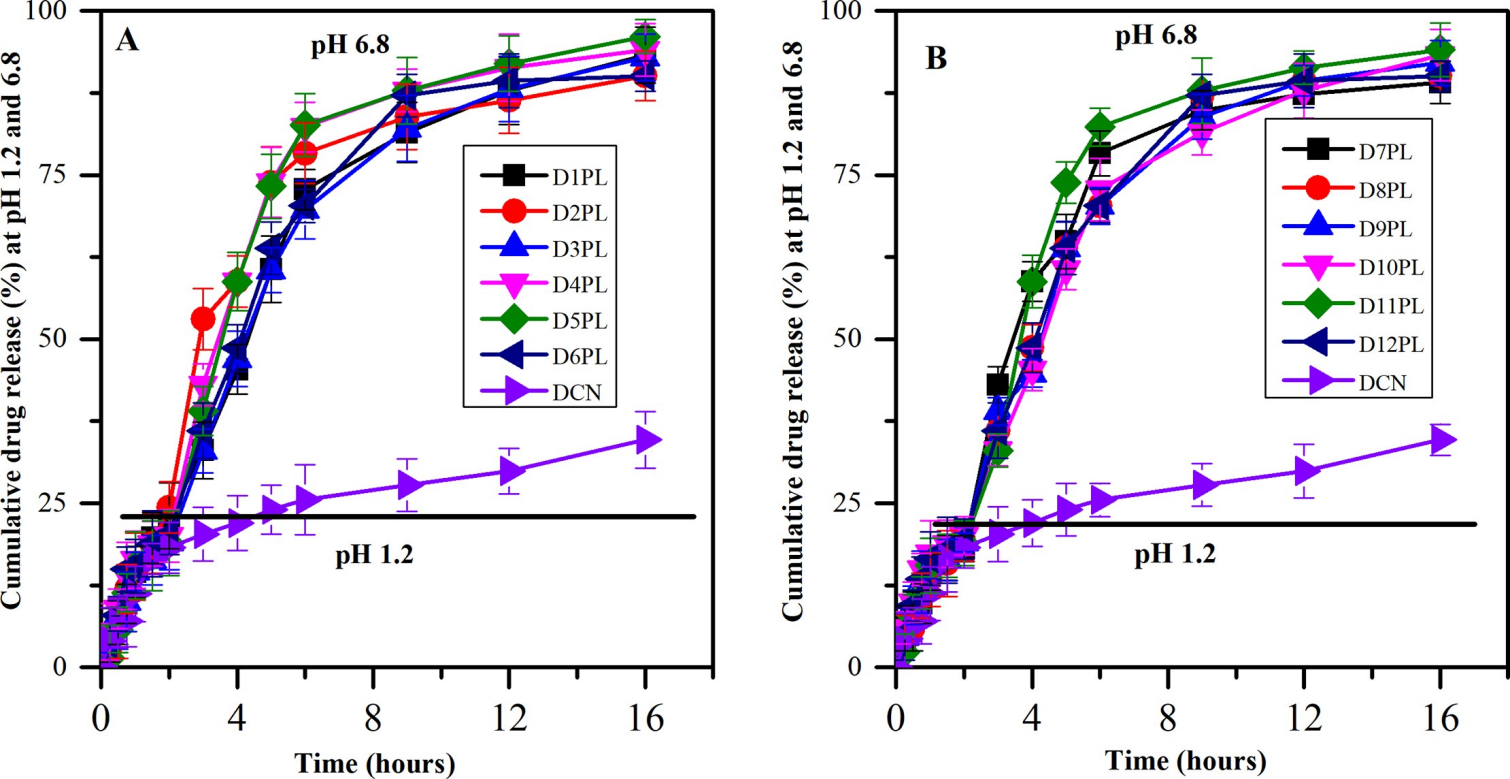
Comparison of drug release profile of PL formulations: A (D1PL-D6PL and drug), and B (D7PL-D12PL and drug).

The *in vitro* release study highlighted that, at simulated gastric pH (pH 1.2) without enzymes, almost 20% of the drug was released from the system within 2 hours. The maximum drug release from the system (>90%) was achieved in 16 hours using a phosphate buffer of pH 6.8 without enzymes as a surrogate indicator of the intestinal medium shown in Fig 5. On the contrary, the diacerein was released 18% in the first two hours at pH 1.2 and 35% in 16 hours at pH 6.8. Similar effects were also reported by Nekkanti, V., et al., 2015 [16]. Initially, all the formulations showed enhanced dissolution as a precursor of enhanced solubility and quick release of the drug from the system due to the presence of hydrophilic carrier (maltodextrin) followed by sustained drug release due to the presence of lipid mixture (lecithin and cholesterol) [36]. The sustained release of the drug might be attributed to the synergistic lipophilic nature of the lipid, cholesterol and diacerein. Among the developed egg lecithin based D5PL formulation represented the highest drug release and likewise, the same trend was also observed in soy lecithin based D11PL formulation. The statistical analysis of *in vitro* release profile of optimized formulations with the individual formulation showed a significant difference (*p*<0.05).

#### 3.2.5. Kinetics of drug release

The regression coefficient (R^2^) is an indicator of the drug release model when it approaches unity. According to the modeling data, the drug release followed first-order release kinetics as the *R*^2^ were in the range of 0.9640–0.9854 (shown in Table 3) which means that the rate of drug release was dependent upon the drug in the liposomes [37]. This phenomenon may also explain the reason for higher drug release from the liposome with higher EE. In the Korsmeyer-Peppas model, the value of release exponent was (0.524–0.630) which means that diacerein was released from liposomes by an anomalous mechanism i.e. non-Fickian diffusion from the system [38].

**Table 3:** Kinetic modeling of drug release profile of PL formulations.

Formulation Code	Zero order	First order	Higuchi model	Korsmeyer-Peppas model	
	*R* ^2^	*R* ^2^	*R* ^2^	*R* ^2^	*n*
D1PL	0.8099	0.9854	0.9302	0.9519	0.616
D2PL	0.6314	0.9798	0.9018	0.9030	0.524
D3PL	0.8221	0.9829	0.9240	0.9501	0.630
D4PL	0.6738	0.9696	0.9025	0.9065	0.546
D5PL	0.7425	0.9640	0.8942	0.9087	0.593
D6PL	0.7718	0.9814	0.9244	0.9393	0.594
D7PL	0.7131	0.9693	0.8991	0.9081	0.571
D8PL	0.7900	0.9781	0.9112	0.9326	0.616
D9PL	0.7922	0.9812	0.9268	0.9452	0.606
D10PL	0.7954	0.9649	0.9353	0.9525	0.602
D11PL	0.6922	0.9806	0.8923	0.8992	0.562
D12PL	0.7704	0.9800	0.9241	0.9388	0.593

*R*^*2*^ = correlation coefficient, *n* = release exponent.

### 3.3. Evaluation of Liposomal gel

#### 3.3.1. Permeation studies by using excised Wistar rat skin

**Fig 6:**
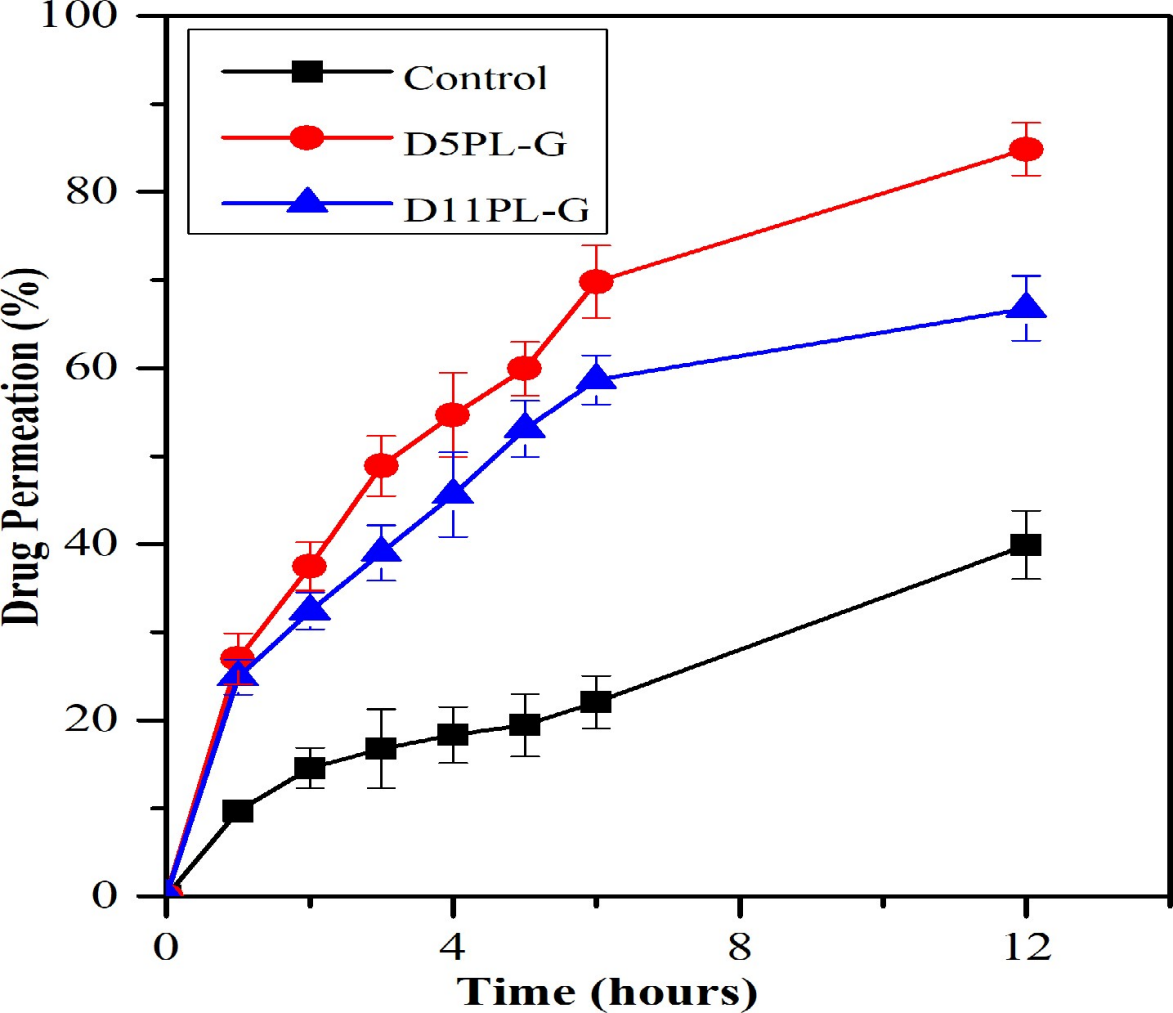
*Ex vivo* permeation analysis of control gel and liposomal gels (D5PL-G and D11PL-G).

Graph of percent drug permeation (μgcm^-2^h^-1^) versus time (hours) was plotted (Fig 6) to evaluate the permeability of selected PL derived liposomal gels (D5PL-G and D11PL-G) and control (drug loaded) gel formulations. The D5PL-G showed an increased value of flux than D11PL-G due to the presence of higher lipid contents in egg lecithin compared to soy lecithin. Likewise, the enhancement ratios of liposomal gels were found 3.50±0.27 (D5PL-G) and 3.21±0.22 (D11PL-G) depicting that the manifold increase in the drug permeation across the skin through PL derived liposomal gel as shown in Table 4. The statistical analysis of permeation studies of liposomal gel with the control gel showed a significant difference (*p*<0.05).

**Table 4:** Permeation analysis of Liposomal gel.

Code	% J (μg/cm^2^hr^-1^)	K_p_ (cm/hr)	E.R
Control	38.15±0.35	4.39±0.26	--
D5PL-G	87.39±0.78	6.93±0.34	3.50±0.27
D11PL-G	69.39±0.67	5.65±0.46	3.21±0.22

All values are expressed as mean ± *SD* (*n* = 3).

### 3.4. Acute oral toxicity study

**Fig 7:**
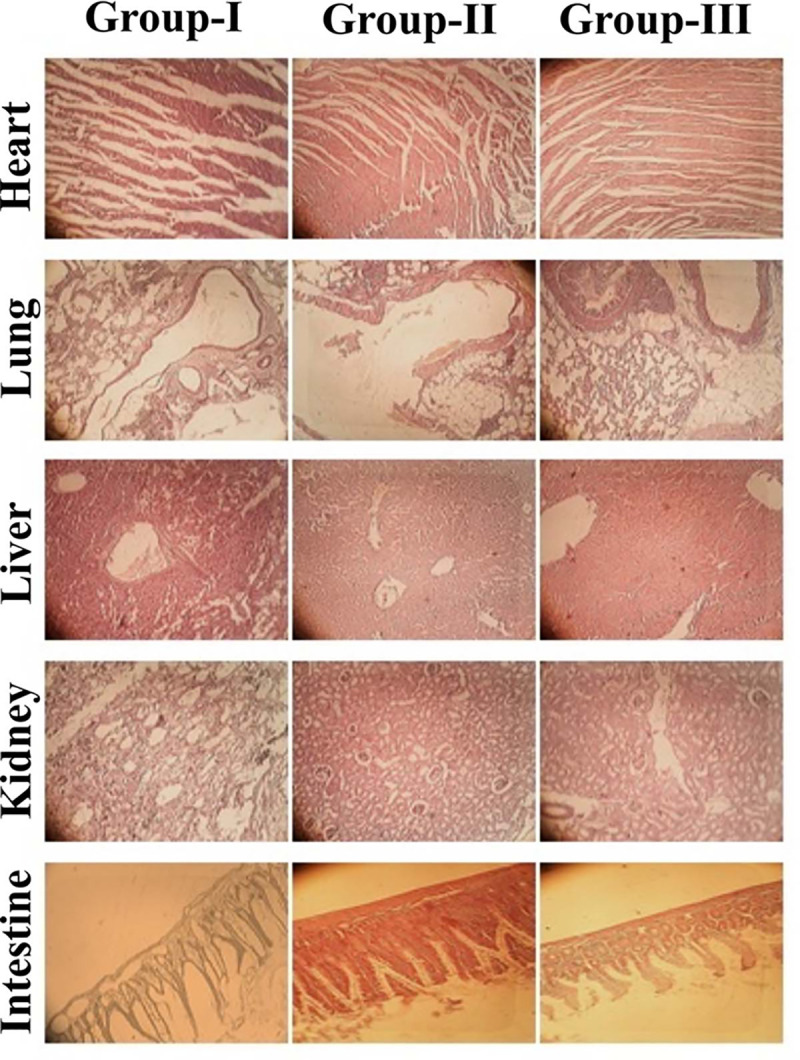
Histopathological microscopic examination of different tissues of Wistar rats of group-I (control), group-II (test) and group-III (test).

Acute oral toxicity study was performed to evaluate liposomal toxicity and biocompatibility in the biological system. No animal was found to be dead during the study, no sign of illness, and any visible skin toxicity/irritation were observed. Wistar rats were sacrificed, and vital organs i.e., heart, lung, liver, kidney and intestine were removed, weighed and dipped in 10% formalin solution after the 14^th^ day. Various parameters of the clinical findings are illustrated in Table 5. The renal, liver function tests and various biochemical parameters showed slight variation as compared to the control group and the overall results were found in line with the already reported literature [28]. No mortality rate was observed during the study, there was neither significant nor gross histopathological lesion in the heart, lung, liver, kidney and intestine tissues of Wistar rats (Fig 7). The lack of change shows the safety and compatibility of the excipients of the PLs [39].

**Table 5:** Biochemical analysis of Wistar rats.

Biochemical analysis	Group I (control)	Group II (test)	Group III (test)
ALT (U/L)	70.33±2.08	69.66±2.88	71.66±2.51
Alkaline Phosphatase (U/L)	499.33±3.05	486.00±3.60	496.33±4.16
Bilirubin (mg/dl)	0.60±0.10	0.63±0.15	0.86±0.05
Urea (mg/dl)	35.33±2.51	36.00±2.64	35.66±2.08
Creatinine (mg/dl)	0.18±0.02	0.21±0.07	0.21±0.21
Uric acid (mg/dl)	2.06±0.15	2.16±-0.20	2.10±0.26
Cholesterol (mg/dl)	60.66±2.08	63.06±2.51	61.00±2.08
Triglycerides (mg/dl)	72.60±1.52	73.66±2.51	75.00±2.00

All values are expressed as mean ± *SD* (*n* = 3).

## 4. Conclusion

The diacerein loaded proliposomes and liposomal derived gels were successfully developed with improved solubility and permeability. The *in vitro* dissolution study substantiated an enhanced solubility of the diacerein. Moreover, the *ex vivo* permeation study displayed the 3.50±0.27 (D5PL-G) and 3.21±0.22 (D11PL-G) folds enhanced permeation across excised Wistar rat skin by egg and soy lecithin based liposomal gels compared to the control. Also, an acute oral toxicity study endorsed that VDDS along with their phospholipids are non-toxic and biocompatible for the biological system. The phospholipids used (egg and soy lecithin) have a comparable effect on the physicochemical characterization of proliposomes, liposomes and liposomal gel whereas, cholesterol has a stabilizing effect on the vesicles. In brief, the developed proliposomes and liposomal gels significantly enhanced the solubility and permeability of diacerein and can provide better management regarding osteoarthritis. In the future, the current study may be extended to the *in vivo* animal study and humans based clinical evaluation for management of osteoarthritis.

## Supporting information

S1 Data (PDF)
