# Supplementary material for: Fabrication, in vitro and ex vivo evaluation of proliposomes and liposomal derived gel for enhanced solubility and permeability of diacerein
Source: PLoS One. 2021 Oct 19;16(10):e0258141. doi: 10.1371/journal.pone.0258141 (PMC8525764; doi:10.1371/journal.pone.0258141)
Supplement: S1 Data — (PDF) [file pone.0258141.s001.pdf]

1. Fourier transform infrared spectroscopic (FTIR) analysis

The FTIR spectra of cholesterol showed characteristic peaks due to stretching at 2930 cm^{-1} (-CH_2 group) and 2865 cm^{-1} (symmetrical –CH_3 group) (1). The characteristic peaks in the FTIR spectrum of maltodextrin were shown at 997 cm^{-1} due to stretching of C-O bond (2). The FTIR spectra of both lipids (egg lecithin and soy lecithin) showed characteristic peaks at 2919 cm^{-1} and 2921 cm^{-1} (due to stretching of -CH of methylene group), and at 1739 cm^{-1} and 1737 cm^{-1} (due to C=O group stretching) as shown in (Fig. S1) (3).

Fig. S1- FTIR analysis of cholesterol (A), maltodextrin (B), soy lecithin (C), and egg lecithin (D).

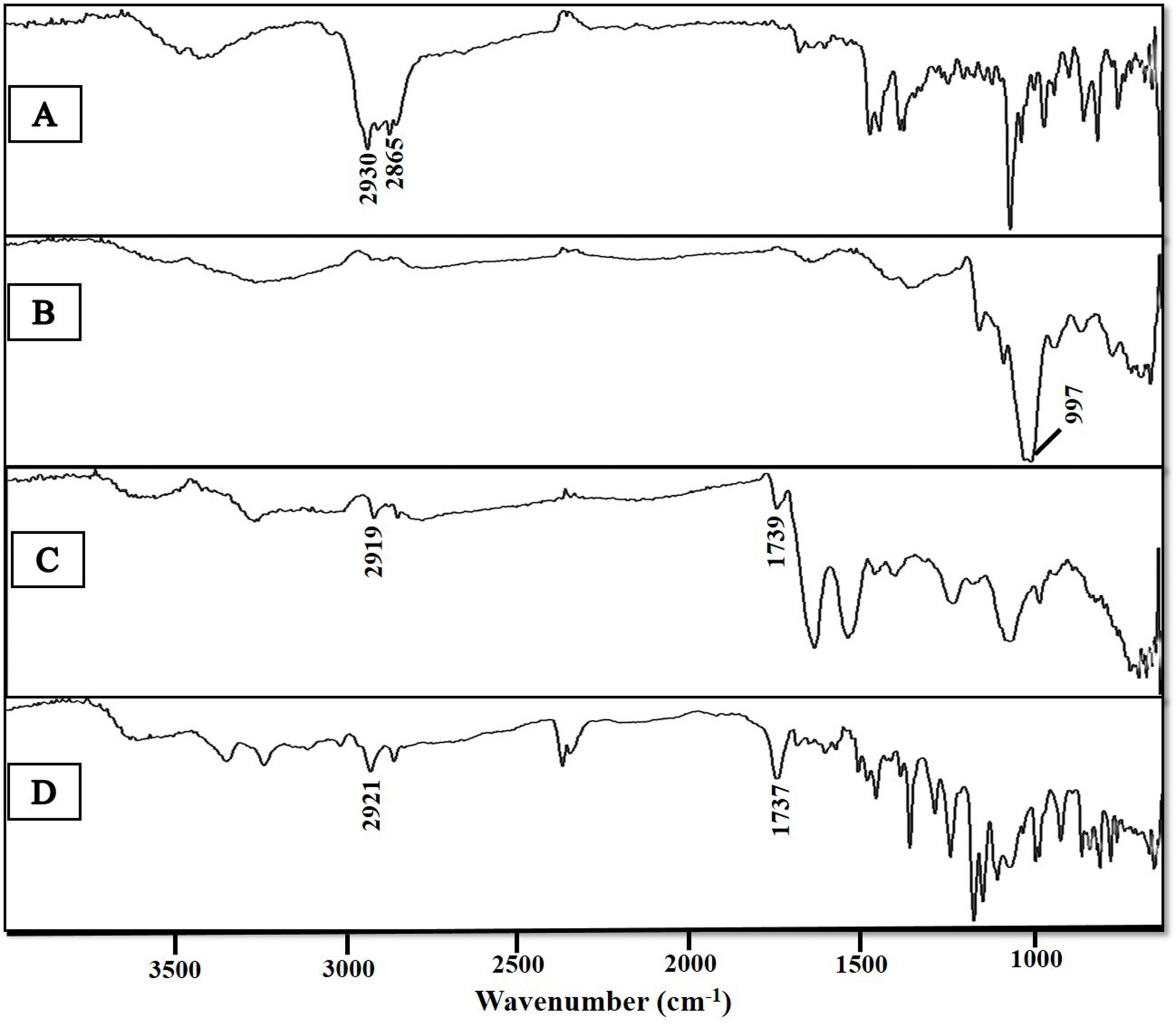
A
2930
2865
B
997
C
2919
1739
D
2921
1737
3500
3000
2500
2000
1500
1000
Wavenumber (cm-1)

2. Thermal stability by differential scanning calorimetric (DSC) analysis

The DSC thermograms of the proliposomal components are mentioned in Fig. S2. The thermograms of cholesterol showed an endothermic peak at 150°C, maltodextrin at 240 °C, egg and soy lecithin in the range of 230-236 °C.

Fig. S2- DSC analysis of cholesterol (A), maltodextrin (B), soy lecithin (C), and egg lecithin (D).

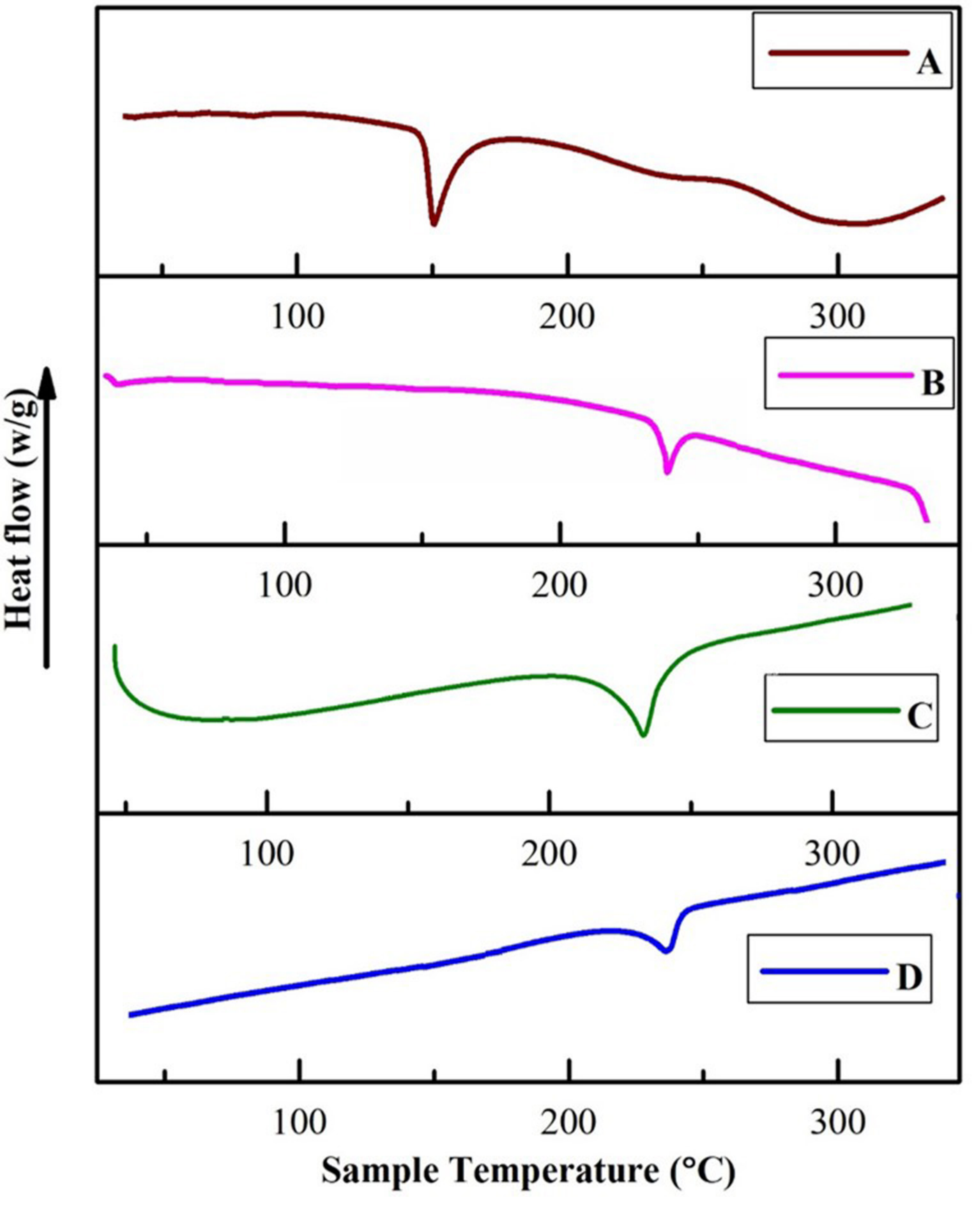

A
B
C
D
100
200
300
Heat flow (w/g)
Sample Temperature (°C)

1. Khan MI, Madni A, Peltonen L. Development and in-vitro characterization of sorbitan monolaurate and poloxamer 184 based niosomes for oral delivery of diacerein. European Journal of Pharmaceutical Sciences. 2016;95:88-95.
2. Castro-Cabado M, Parra-Ruiz FJ, Casado A, Román JS. Thermal crosslinking of maltodextrin and citric acid. Methodology to control the polycondensation reaction under processing conditions. Polymers and polymer composites. 2016;24(8):643-54.
3. Wang X, Luo Z, Xiao Z. Preparation, characterization, and thermal stability of β-cyclodextrin/soybean lecithin inclusion complex. Carbohydrate polymers. 2014;101:1027-32.
